# Condensin II Resolves Chromosomal Associations to Enable Anaphase I Segregation in *Drosophila* Male Meiosis

**DOI:** 10.1371/journal.pgen.1000228

**Published:** 2008-10-17

**Authors:** Tom A. Hartl, Sarah J. Sweeney, Peter J. Knepler, Giovanni Bosco

**Affiliations:** Department of Molecular and Cellular Biology, University of Arizona, Tucson, Arizona, United States of America; Stowers Institute for Medical Research, United States of America

## Abstract

Several meiotic processes ensure faithful chromosome segregation to create haploid gametes. Errors to any one of these processes can lead to zygotic aneuploidy with the potential for developmental abnormalities. During prophase I of *Drosophila* male meiosis, each bivalent condenses and becomes sequestered into discrete chromosome territories. Here, we demonstrate that two predicted condensin II subunits, Cap-H2 and Cap-D3, are required to promote territory formation. In mutants of either subunit, territory formation fails and chromatin is dispersed throughout the nucleus. Anaphase I is also abnormal in *Cap-H2* mutants as chromatin bridges are found between segregating heterologous and homologous chromosomes. Aneuploid sperm may be generated from these defects as they occur at an elevated frequency and are genotypically consistent with anaphase I segregation defects. We propose that condensin II–mediated prophase I territory formation prevents and/or resolves heterologous chromosomal associations to alleviate their potential interference in anaphase I segregation. Furthermore, condensin II–catalyzed prophase I chromosome condensation may be necessary to resolve associations between paired homologous chromosomes of each bivalent. These persistent chromosome associations likely consist of DNA entanglements, but may be more specific as anaphase I bridging was rescued by mutations in the homolog conjunction factor *teflon*. We propose that the consequence of condensin II mutations is a failure to resolve heterologous and homologous associations mediated by entangled DNA and/or homolog conjunction factors. Furthermore, persistence of homologous and heterologous interchromosomal associations lead to anaphase I chromatin bridging and the generation of aneuploid gametes.

## Author Summary

Some of the processes that ensure proper chromosome segregation take place upon the chromosomes themselves. The chromosomes of *Drosophila* males undergo an interesting and relatively enigmatic step before entering meiosis, where each paired homologous chromosome becomes clustered into a discrete region of the nucleus. In this article, we provide evidence that improper chromosomal associations are resolved and/or prevented during this “chromosome territory” formation. This was uncovered through the study of flies mutant for *Cap-H2*, which have abnormal territory formation and improper chromosomal associations that persist into segregation. Another important process that chromosomes undergo in meiosis is the pairing and physical linking of maternal and paternal homologs to one another. Linkages between homologs are essential to ensure their proper segregation to daughter cells. In contrast to meiosis in most organisms, linkages between homologs in male *Drosophila* are not recombination mediated. Here, we provide evidence that Cap-H2 may function to remove *Drosophila* male specific linkages between homologous chromosomes prior to anaphase I segregation. When chromosomal associations persist during segregation of *Cap-H2* mutants, the chromosomes do not detach from one another and chromatin is bridged between daughter nuclei. The likely outcome of this defect is the production of aneuploid sperm.

## Introduction

There are several critical steps that chromosomes must undergo as they transition from their diffuse interphase state to mobile units that can be faithfully transmitted to daughter cells. In the germline, faulty segregation leading to the creation of aneuploid gametes is likely a leading cause of genetic disease, miscarriages, and infertility in humans [1].

Some steps that promote proper segregation are universal to all cell types undergoing cell division. Chromosomal “individualization” is necessary to remove DNA entanglements that likely become introduced naturally through movements of the threadlike interphase chromatin [2]. Topoisomerase II (top2) contributes to individualization with its ability to pass chromosomes through one another by creating and resealing double strand breaks [3]. The necessity of top2's “decatenation” activity to chromosome individualization becomes clear from fission yeast *top2* mutants and vertebrate cells treated with a top2 inhibitor, where mitotic chromosomes appear associated through DNA threads [4],[5]. Another step that occurs prior to chromosome segregation is chromosome “condensation,” entailing the longitudinal shortening from the threadlike interphase state into the rod like mitotic chromosome [2]. Condensation is necessary due to the great linear length of interphase chromosomes that would be impossible to completely transmit to daughter cells.

Because chromosome individualization and condensation appear to occur concurrently, it has been inferred that both are promoted by the same catalytic activity. In support of this idea, the condensin complexes have been implicated in chromosome individualization [6] and condensation [7], suggesting a molecular coupling of both processes. The condensin I and II complexes are thought to be conserved throughout metazoa, each utilizing ATPases SMC2 and SMC4, but carrying different non-SMC subunits Cap-H, Cap-G, Cap-D2 or Cap-H2, Cap-G2, and Cap-D3, respectively [7]–[9]. *In vitro*, condensin I is known to induce and trap positive supercoils into a circular DNA template [10]–[12]. Current models to explain condensin I chromosome condensation highlight this activity as supercoiling may promote chromatin gathering into domains that can then be assembled into a higher order structure [13]. Condensin complexes may also promote condensation and individualization through cooperating with other factors, such as chromatin-modifying enzymes [14]–[17] and top2 [15], [18]–[22]. While the effect of condensin mutations or RNAi knockdown on chromosome condensation is variable depending on cell type and organism being studied, in most if not all cases, chromatin bridges are created between chromosomes as they segregate from one another [7]. This likely represents a general role of the condensin complex in the resolution of chromosomal associations prior to segregation.

While the second cell division of meiosis is conceptually similar to mitotic divisions where sister chromatids segregate from one another, the faithful segregation of homologous chromosomes in meiosis I requires several unique steps. It is essential for homologous chromosomes to become linked to one another for proper anaphase I segregation [23] and most often this occurs through crossing over to form chiasmata [24]. As recombination requires the close juxtaposition of homologous sequences, homologs must first “identify” one another in the nucleus and then gradually become “aligned” in a manner that is DNA homology dependent, but not necessarily dictated by the DNA molecule itself. Eventually, the homologous chromosomes become “paired,” which is defined as the point when intimate and stable associations are established. The paired state is often accompanied by the laying down of a proteinaceous structure called the synaptonemal complex between paired homologous chromosomes, often referred to as “synapsis” [25],[26]. Importantly, the recombination mediated chiasmata can only provide a linkage between homologs in cooperation with sister chromatid cohesion distal to the crossover [27].

*Drosophila* male meiosis is unconventional in that neither recombination [28] nor synaptonemal complex formation occur [29], yet homologous chromosomes still faithfully segregate from one another in meiosis I. Two proteins have been identified that act as homolog pairing maintenance factors and may serve as a functional replacement of chiasmata. Mutations to genes encoding these achiasmate conjunction factors, *MNM* and *SNM*, cause homologs to prematurely separate and by metaphase I, they can be observed as univalents that then have random segregation patterns. It is likely that MNM and SNM directly provide conjunction of homologs as both localize to the X–Y pairing center (rDNA locus) up until anaphase I and an MNM-GFP fusion parallels this temporal pattern at foci along the 2^nd^ and 3^rd^ chromosomes [30]. While MNM and SNM are required for the conjunction of all bivalents, the protein Teflon promotes pairing maintenance specifically for the autosomes [31],[32]. Teflon is also required for MNM-GFP localization to the 2^nd^ and 3^rd^ chromosomes [30]. This suggests that Teflon, MNM, and SNM constitute an autosomal homolog pairing maintenance complex.

A fascinating aspect of *Drosophila* male meiosis is that during prophase I, three discrete clusters of chromatin become sequestered to the periphery of the nuclear envelope's interior. Each of these “chromosome territories” corresponds to one of the major chromosomal bivalents, either the 2^nd^, 3^rd^ or X–Y [33]–[36]. A study of chromosomal associations within each prophase I bivalent demonstrated that the four chromatids begin in close alignment. Later in prophase I, all chromatids seemingly separate from one another, but the bivalent remains intact within the territory [36]. It has therefore been proposed that chromosome territories may provide stability to bivalent associations through their sequestration into sub-nuclear compartments [36].

Here we document that *Drosophila* putative condensin II complex subunits, Cap-H2 and Cap-D3, are necessary for normal territory formation. When they are compromised through mutation, chromatin is seemingly dispersed throughout the nucleus. We propose that the consequence of this defect is failure to individualize chromosomes from one another leading to the introduction and/or persistence of heterologous chromosomal associations into anaphase I. This underscores the role of chromosome territory formation to prevent ectopic chromosomal associations from interfering with anaphase I segregation. Cap-H2 is also necessary to resolve homologous chromosomal associations, that like heterologous associations, may be mediated by DNA entanglements and/or persistent achiasmate conjunction as anaphase I bridging is rescued by *teflon* mutations. This highlights condensin II mediated chromosome individualization/disjunction in meiosis I and its necessity to the creation of haploid gametes.

## Results/Discussion

### The Predicted Condensin II Subunits Cap-H2 and Cap-D3 Are Necessary for Male Fertility

Faithful chromosome segregation is necessary to organismal viability, therefore it is not surprising that in *Drosophila*, homozygous lethal alleles exist in the following condensin subunits: SMC4/gluon, SMC2, Cap-H/barren, and Cap-G [19],[37],[38]. It has however been reported that one mutant *Cap-D3* allele, *Cap-D3^EY00456^* (Figure S1) is homozygous viable, yet completely male sterile [39]. We have confirmed the necessity of *Cap-D3* to male fertility as both *Cap-D3^EY00456^* homozygous and *Cap-D3^EY00456^*/*Cap-D3^Df(2L)Exel6023^* males were completely sterile when mated to wild-type females. Furthermore, males *trans*-heterozygous for strong *Cap-H2* mutations (Figure S1) were also male sterile as no progeny were derived from crosses of *Cap-H2^Z3-0019^/Cap-H2^Df(3R)Exel6159^*, *Cap-H2^TH1^/Cap-H2^Df(3R)Exel6159^*, and *Cap-H2^TH1^/Cap-H2^Z3-0019^* to wild-type females. A third allele, *Cap-H2^Z3-5163^* (Figure S1), was found to be fertile as a homozygote and in *trans*-combinations with *Cap-H2^Z3-0019^*, *Cap-H2^Df(3R)Exel6159^*, and *Cap-H2^TH1^* alleles.

**Figure 1:**
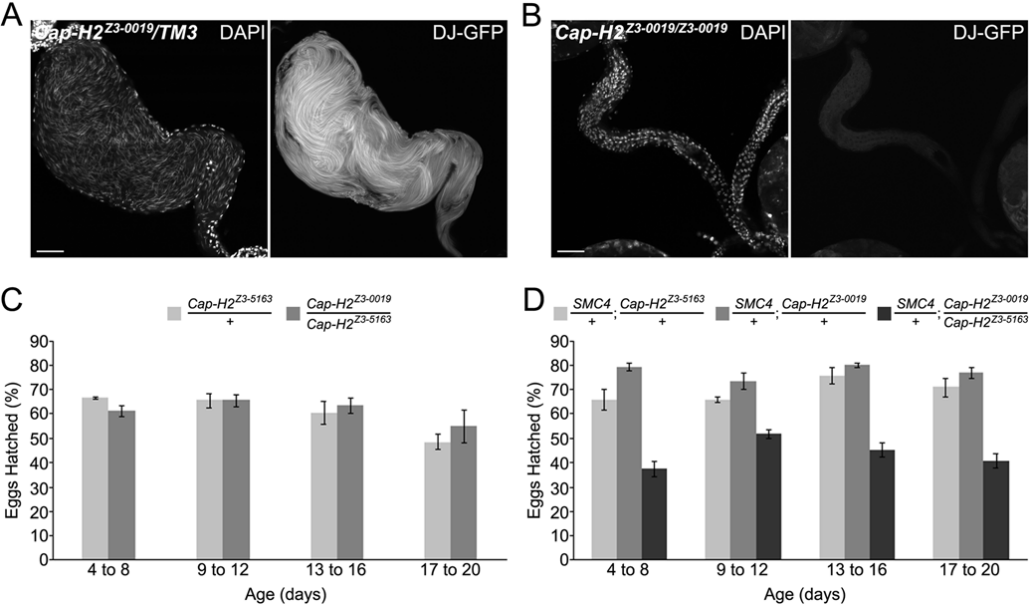
*Cap-H2* allelic combinations range from completely male sterile to only having detectable fertility loss when also heterozygous for an *SMC4* mutation. (A) Seminal vesicle from *Cap-H2* heterozygous male. The abundance of sperm is evident through the visualization of sperm heads (DAPI) and tails (don juan-GFP). Scale bar indicates 50 µm. (B) Seminal vesicle from a male sterile *Cap-H2* mutant. DAPI and don juan-GFP illustrate the absence of mature sperm. (C) *Cap-H2^Z3-0019^/Cap-H2^Z3-5163^* males had a fertility equivalent to *Cap-H2^Z3-5163^/+* controls (*p*>0.05, two-tailed T-test assuming equal variances). Males were mated to wild-type females and the percent of eggs hatched was quantified. Data for each timepoint represent the same set of males with a different brood of females. (D) The introduction of a mutant *SMC4* allele into the *Cap-H2^Z3-0019^/Cap-H2^Z3-5163^* mutant background reduced fertility compared to *SMC4*; *Cap-H2* double heterozygous controls (*p*<0.05, two-tailed T-test assuming equal variances). No other pairwise comparison was found to be significant (*p*>0.05).

To determine whether the primary defect leading to loss of fertility in *Cap-H2* mutant males is pre or post copulation, *Cap-H2^Z3-0019^* homozygous mutant and heterozygous control siblings were engineered to carry a sperm tail marker, *don juan-GFP*, and aged in the absence of females to allow sperm to accumulate in the seminal vesicles. In contrast to *Cap-H2^Z3-0019^* heterozygous control males where the seminal vesicles fill with sperm, those from *Cap-H2^Z3-0019^* homozygous males were seemingly devoid of sperm as no DAPI staining sperm heads or *don juan-GFP* positive sperm tails were detectable (Figure 1A and 1B). The lack of mature sperm in the seminal vesicles confirmed that sterility in *Cap-H2* mutant males is attributed to a defect in gamete production.

To test whether a *Cap-H2* mutant allelic combination that is male fertile, *Cap-H2^Z3-0019^*/*Cap-H2^Z3-5163^*, has a decreased fertility, males of this genotype and heterozygous controls were mated to wild-type females and the percent of eggs hatched was quantified. There was no significant difference in male fertility between *Cap-H2^Z3-0019^*/*Cap-H2^Z3-5163^* and *Cap-H2^Z3-5163^*/+ males (Figure 1C). However, the introduction of one mutant allele of another condensin subunit, *SMC4^08819^*, to the *Cap-H2 trans*-heterozygote led to a substantial decrease in fertility relative to the *SMC4^08819^*/+; *Cap-H2^Z3-5163^*/+ and *SMC4^08819^*/+; *Cap-H2^Z3-0019^/+* double heterozygous controls (Figure 1D). This suggests that Cap-H2 is functioning in the *Drosophila* male germline as a member of a condensin complex along with SMC4 during gametogenesis.

### Male fertile Cap-H2 Allelic Combinations Lead to 2^nd^ and 3^rd^ Nondisjunction, but Normal 4^th^ and Sex Chromosome Segregation

Given the well-documented roles of condensin subunits in promoting chromosome segregation [7], we reasoned that a possible cause of fertility loss in *Cap-H2* and *Cap-D3* mutants is through chromosome missegregation in the male germline. Male gametogenesis begins with a germline stem cell division. While one daughter maintains stem cell identity, the gonialblast initiates a mitotic program where 4 synchronous cell divisions create a cyst of 16 primary spermatocytes that remain connected due to incomplete cytokinesis. These mature over a period of 3.5 days, undergo DNA replication, and subsequently enter meiosis [34]. To test whether chromosome segregation defects occur during gametogenesis of *Cap-H2* mutants, i.e. during the mitotic divisions of the stem cell or gonia or from either meiotic divisions, genetic tests were performed that can detect whether males create an elevated level of aneuploid sperm. In these “nondisjunction” assays, males are mated to females that have been manipulated to carry a fused, or “compound”, chromosome. Females bearing a compound chromosome and specific genetic markers are often necessary to determine whether eggs had been fertilized by aneuploid sperm. Importantly, in nondisjunction assays, fertilizations from aneuploid sperm generate “exceptional” progeny that can be phenotypically distinguished from “normal” progeny that were created from haploid sperm fertilizations.

Sex chromosome segregation was monitored as previously described for mutants in the *ord* gene [40], with males bred to carry genetic markers on the X and Y chromosomes. These *y^1^w^1^*/*y*+Y; *Cap-H2^Z3-0019^*/*Cap-H2^Z3-5163^* and corresponding *Cap-H2* heterozygous controls males were crossed to females bearing compound X chromosomes (*C(1)RM*, *y^2^ su(w^a^)w^a^*). As shown in Table 1, no significant amount of exceptional progeny were generated from *Cap-H2* mutant males. It is important to point out that the lack of significant sex chromosome segregation defects found in these nondisjunction assays with a likely weak *Cap-H2* male fertile mutant may be misleading. In fact, sex chromosome segregation defects are observed cytologically in stronger *Cap-H2* mutant backgrounds that could not be tested with nondisjunction assays because of their sterility (see below).

**Table 1:** Sex chromosome nondisjunction was not found in *Cap-H2* fertile males.

Paternal Genotype		Regular Sperm		Exceptional Sperm				
Sex	Chr. 3	X	Y(Y)	nullo-XY	XY(Y)	XX	XXY(Y)	Total Progeny
*y^1^w^1^/y+Y*	*Cap-H2^Z3-0019^/Cap-H2^Z3-5163^*	160	165	1	0	0	0	326
*y^1^w^1^/y+Y*	*Cap-H2^Z3-0019^/TM3, Ser*	179	129	0	0	0	0	308
*y^1^w^1^/y+Y*	*Cap-H2^Z3-5163^/TM3, Ser*	132	151	0	0	0	0	283
*y^1^w^1^/y+Y*	*Cap-H2^Z3-5163^/Cap-H2^TH1^*	227	160	1	0	0	0	388
*y^1^w^1^/y+Y*	*Cap-H2^TH1^/TM3, Sb*	215	184	0	0	0	0	399

Fourth chromosome segregation was assayed as described previously for *teflon* mutants [32], with males carrying one copy of a 4^th^ chromosome marker mated to females bearing compound 4^th^ chromosomes (*C(4)EN*, *ci ey*). As with the sex chromosome segregation assays, 4^th^ chromosome segregation did not differ substantially between the *Cap-H2^Z3-0019^*/*Cap-H2^Z3-5163^* and heterozygous control males (Table 2). The possibility remains that this hypomorphic *Cap-H2* allelic combination is not strong enough to reveal 4^th^ chromosome segregation defects. Like sex chromosomes, 4^th^ chromosome segregation abnormalities were observed cytologically in stronger male sterile mutants (see below).

**Table 2:** 4^th^ chromosome nondisjunction was not found in *Cap-H2* fertile males.

Paternal Genotype		haplo-4 (*spa^pol^* or +)	diplo-4 (*spa^pol^/spa^pol^*)	nullo-4	total	% 4^th^ NDJ
Chr 3	Chr. 4					
*Cap-H2^Z3-0019^/Cap-H2^Z3-5163^*	*spa^pol^/+*	806	1	2	809	0.37
*Cap-H2^Z3-0019^/TM6B, Hu, Tb^3^*	*spa^pol^/+*	298	1	0	299	0.33
*Cap-H2^Z3-5163^/TM6B, Hu, Tb*	*spa^pol^/+*	338	0	0	338	0.00

Effects on second and third chromosome segregation were assayed with the use of females carrying either compound 2 (*C(2)EN*, *b pr*) or compound 3 (*C(3)EN*, *st cu e*) chromosomes. Interestingly, both the 2^nd^ and 3^rd^ chromosomes had a heightened sensitivity to *Cap-H2* mutation as *Cap-H2^Z3-0019^*/*Cap-H2^Z3-5163^* males created an elevated level of exceptional progeny (Tables 3 and 4). In both cases, the exceptional class most over represented were those from fertilization events involving sperm that lacked a 2^nd^ (nullo-2) or 3^rd^ (nullo-3) chromosome.

**Table 3:** 2^nd^ chromosome nondisjunction is elevated in *Cap-H2* fertile males.

Paternal Genotype		n	nullo-2	diplo-2 (*bw/*+)	diplo-2 (*bw/bw*)	diplo-2 (+/+)	Total Progeny
Chr. 2	Chr. 3						
*bw/+*	*Cap-H2^Z3-0019^/Cap-H2^Z3-5163^*	190	0.11 (20)	0.08 (16)	0.05 (10)	0.01 (2)	0.26 (50)
*bw/+*	*Cap-H2^Z3-0019^/+*	120	0.03 (4)	0.1 (12)	0.04 (5)	0 (0)	0.18 (21)
*bw/+*	*Cap-H2^Z3-5163^/+*	200	0 (0)	0.05 (10)	0.04 (8)	0 (0)	0.09 (18)
*bw/+*	*+/+*	150	0.05 (8)	0.05 (7)	0.02 (3)	0.01 (1)	0.13 (19)

“n” refers to the number of males tested. Two values are displayed in the progeny class columns. The first value represents the ratio of progeny to male tested. The second is the number of progeny for that particular class.

**Table 4:** 3^rd^ chromosome nondisjunction is elevated in *Cap-H2* fertile males.

Paternal Genotype	n	nullo-3	diplo-3 (het)^1^	diplo-3 (homo)^2^	ND^3^	Total progeny
*Cap-H2^Z3-0019^/Cap-H2^Z3-5163^*	30	24	1	0	4	29
*Cap-H2^Z3-5163^/Cap-H2^Z3-5163^*	30	16	1^4^	1^4^	0	17
*Cap-H2^Z3-0019^/+*	30	2	1	0	0	3
*Cap-H2^Z3-5163^/+*	30	1	0	0	2	3
*+/+*	30	1	0	0	1	2

“n” refers to the number of males tested.

1 Diplo-3 (het) refers to sperm that were heterozygous for the paternal male's 3^rd^ chromosome loci.

2 Diplo-3 (homo) sperm were homozygous for the paternal male's third chromosome loci.

3 ND refers to a class of F1 progeny with genotypes that were “not determinable” because the flies died before they could be test crossed or the markers of the F2 progeny could not distinguish between classes.

4 In the testing of the *Cap-H2^Z3-5163^/Cap-H2^Z3-5163^* males, diplo-3 (het) and diplo-3 (homo) progeny were indistinguishable because they are genotypically identical for third chromosome loci.

Nullo progeny can be created from defects in either meiotic division. For example, the reciprocal event of incorrect cosegregation of homologs during meiosis I is one daughter cell completely lacking that particular chromosome. Similarly, nullo sperm can be created from meiosis II defects where sister chromatids cosegregate. To address whether meiotic I and or II segregation defects occur, males in the 2^nd^ chromosome assays were bred to be heterozygous for the 2^nd^ chromosome marker *brown* (*bw^1^*). If both 2^nd^ homologous chromosomes mistakenly cosegregate in meiosis I, then a normal meiosis II will generate diplo-2 sperm that are heterozygous for the paternal male's 2^nd^ chromosomes (*bw^1^/+*). Additionally, a normal meiosis I followed by a faulty meiosis II where sister chromatids cosegregate would generate diplo-2 sperm homozygous for the paternal male's 2^nd^ chromosomes (*bw^1^/bw^1^* or +/+). There was a trend toward an elevated level of the *bw^1^/+* exceptional class from both *Cap-H2^Z3-0019^*/*Cap-H2^Z3-5163^* and *Cap-H2^Z3-0019^*/+ males. This suggested meiosis I nondisjunction that possibly occurs even in *Cap-H2* heterozygous males. Furthermore, there may also be a slight increase in meiosis II nondisjunction as the *bw^1^/bw^1^* class is elevated in the *Cap-H2 trans*-heterozygous and heterozygous males.

The *Cap-H2* allelic combination utilized in these genetic nondisjunction assays is likely weak in comparison to others where males are completely sterile. Therefore, the elevated frequency of exceptional progeny from 2^nd^ and 3^rd^ chromosome assays relative to the sex and 4^th^ may only represent a heightened sensitivity of these chromosomes rather then a role for Cap-H2 specifically in 2^nd^ and 3^rd^ chromosome segregation. In fact, defects in sex and 4^th^ chromosome segregation were observed in stronger male sterile *Cap-H2* mutants (see below). One possible explanation for a major autosome bias in our nondisjunction assays may be related to the greater amount of DNA estimated for the 2^nd^ (60.8 Mb) and 3^rd^ (68.8 Mb) relative to the X, Y, and 4^th^ chromosomes (41.8, 40.9, and 4.4 Mb, respectively) [41]. Thus, perhaps larger chromosomes require more overall condensin II function to promote their individualization or condensation and are therefore more sensitive to Cap-H2 dosage. While plausible, if sensitivity to Cap-H2 mutation were purely due to chromosome size, it is difficult to explain why a more significant level of XY nondisjunction did not occur given that they are ∼70% the size of the 2^nd^ and 3^rd^.

An alternative hypothesis involves the fact that 2^nd^ chromosome conjunction may occur at several sites or along its entire length [42], whereas XY bivalent pairing is restricted to intergenic repeats of the rDNA locus [43],[44]. This suggests that more total DNA is utilized for conjunction of the 2^nd^ chromosome relative to the sex bivalent. Assuming the 3^rd^ and 4^th^ chromosomes maintain homolog pairing like the 2^nd^, then the relative amount of DNA utilized in conjunction is as follows: 3^rd^>2^nd^>4^th^>XY. Given that this closely parallels the trend of sensitivity to *Cap-H2* mutation in the nondisjunction assays, it suggests that chromosomes which utilize more overall DNA in pairing/pairing maintenance activities require a greater dose of functional Cap-H2 for their proper anaphase I segregation. This points toward a role for Cap-H2 in the regulation of homolog conjunction/disjunction processes. We next addressed this hypothesis through cytological analyses of meiotic chromosome morphology in *Cap-H2* mutant backgrounds.

### Cap-H2 Mutants Are Defective in Prophase I Chromosome Territory Formation

**Figure 2:**
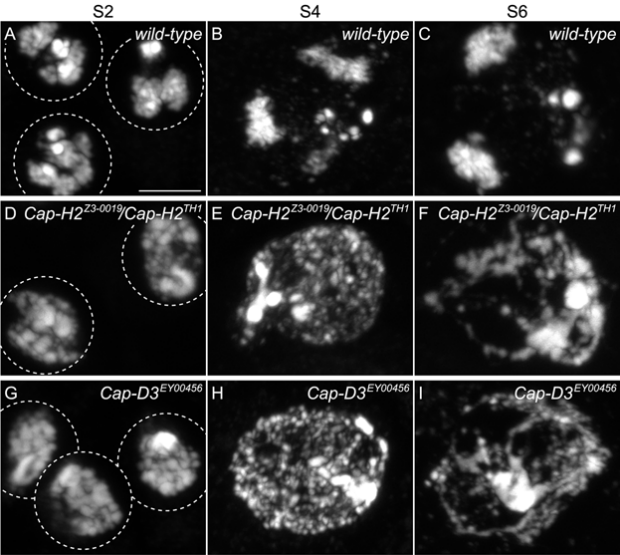
Male sterile *Cap-H2* mutants have irregular territory formation throughout prophase I. Three wild-type early prophase I (S2) primary spermatocyte nuclei stained with DAPI. Chromatin clustering is likely an early indicator of chromosome territory formation. Scale bar indicates 5 µm and serves all panels. (B) Mid wild-type prophase I (S4) nucleus where territory formation becomes more clear as indicated by three DAPI staining regions. (C) Late prophase I (S6) wild-type nucleus where the three DAPI staining chromosome territories are prominent. (D) Two prophase I (S2) nuclei from *Cap-H2^Z3-0019^/Cap-H2^TH1^* mutants displaying abnormal chromatin organization that likely represents failure in the early stages of chromosome territory formation. This phenotype is 100% penetrant as it was observed in all S2 nuclei (n = 100). (E) Mid Prophase I (S4) nucleus from a *Cap-H2^Z3-0019^/Cap-H2^TH1^* male where chromosome territory formation fails and instead chromatin appears throughout the nucleus. All S4 nuclei observed were similarly defective (n = 100). (F) Late prophase I (S6) nucleus from a *Cap-H2^Z3-0019^/Cap-H2^TH1^* male where no discrete chromosome territories can be observed. All S6 nuclei observed had a similar abnormal morphology (n = 100). (G) Three early prophase I (S2) nuclei from a *Cap-D3^EY00456^* male where chromosome morphology is abnormal and likely represents failure in the early stages of territory formation. All S2 nuclei observed were similarly defective (n = 100). (H) Mid prophase I (S4) nucleus from a *Cap-D3^EY00456^* male where chromosome territory formation fails and instead chromatin appears throughout the nucleus. All S4 nuclei observed carried a similarly abnormal chromosome organization (n = 100). (I) Late prophase I (S6) nucleus from a *Cap-D3^EY00456^* mutant male where discrete territory formation is absent. All S6 nuclei were similarly defective (n = 100).

In prophase I stage S2 (Figure 2A), nuclei appear to commence the formation of chromosome territories. By mid-prophase I stage S4, territory formation is more evident (Figure 2B) and in late prophase I, stage S6 nuclei exhibit three discrete chromosome territories seemingly associated with the nuclear envelope (Figure 2C) [35]. Each of the three chromosome territories corresponds to the 2^nd^, 3^rd^, and sex chromosomal bivalents and are thought to have important chromosome organizational roles for meiosis I [33]–[36]. In male sterile mutants of the genotype *Cap-H2^Z3-0019^*/*Cap-H2^TH1^*, chromosome organizational steps throughout prophase I are defective, as normal territory formation is never observed in 100% of S2, S4, and S6 stages (n = 100 nuclei of each stage). Instead, chromatin is seemingly dispersed within the nucleus (Figures 2D–2F). Male sterile *Cap-D3^EY00456^* mutants mimic these defects (Figure 2G–2I), suggesting that Cap-D3 and Cap-H2 function together within a condensin II complex to facilitate territory formation. No prophase I defects were observed in *Cap-H2^Z3-0019^*/*Cap-H2^Z3-5163^* males, although subtle morphological changes may be difficult to detect.

To establish possible roles for Cap-H2 and Cap-D3 in prophase I chromosome organization, it is important to outline the two general processes that must occur for proper territory formation. One is to gather or condense bivalent chromatin into an individual cluster. The second is to sequester each bivalent into a discrete pocket of the nucleus. Condensin II may perform one or both tasks, for example, perhaps chromatin is dispersed throughout the nucleus in the *Cap-H2/Cap-D3* mutants because of faulty condensation. Alternatively, or in addition to, sequestration of chromatin into territories may be a primary defect in *Cap-H2/Cap-D3* mutants.

### Cap-H2 and Cap-D3 Resolve Chromosomal Associations Prior to Anaphase I

**Figure 3:**
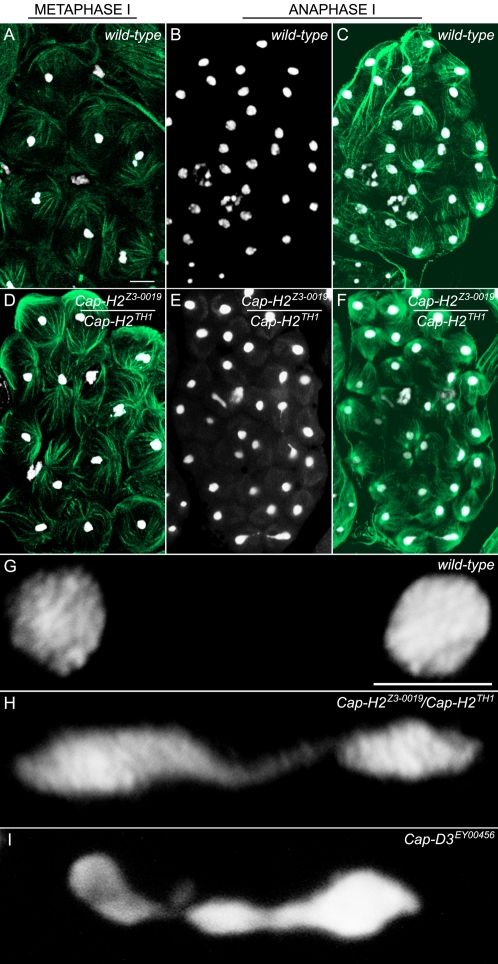
Chromosomes remain associated into anaphase I of *Cap-H2* mutants. Metaphase I and anaphase I morphologies were compared between wild-type and *Cap-H2^Z3-0019^/Cap-H2^TH1^* mutant males. Testes were stained with DAPI and an anti-tubulin antibody to visualize DNA (white) and microtubules (green), respectively (scale bar in 3A indicates 10 µm and 5 µm in 3G). (A) Metaphase I in the wild-type. Each bivalent has congressed to the metaphase plate and appears as a cluster of DAPI stained material. (B) Anaphase I in the wild-type (DAPI only). Homologous chromosomes have segregated to daughter cells. (C) Anaphase I in the wild-type (DAPI and Tubulin merge). (D) Metaphase I from a *Cap-H2^Z3-0019^/Cap-H2^TH1^* mutant male appears wild-type. (E) Anaphase I from a *Cap-H2^Z3-0019^/Cap-H2^TH1^* mutant male (DAPI only). Chromatin bridges can be seen in three different segregation events. (F) Anaphase I from a *Cap-H2^Z3-0019^/Cap-H2^TH1^* mutant male (DAPI and Tubulin merge). (G) Higher resolution wild-type anaphase I image showing complete segregation of homologs. (H) Anaphase I from a *Cap-H2^Z3-0019^/Cap-H2^TH1^* mutant demonstrating extensive chromatin bridging due to persistent associations between chromosomes migrating to opposing poles. (I) Anaphase I bridge found from a *Cap-D3^EY00456^* mutant.

During late prophase I of wild-type primary spermatocytes, chromosomes from each territory condense further and appear as three dots corresponding to the 2^nd^, 3^rd^ and sex bivalents. This stage, referred to as M1 of meiosis I, may be morphologically abnormal in strong *Cap-H2* mutants because it was not detected in our studies (n>50 testes). This is likely because these mutants fail to form normal chromosome territories. Proceeding further into meiosis, metaphase I is signified by the congression of the three bivalents into one cluster at the metaphase plate (Figure 3A). Despite not forming normal chromosome territories and possibly never reaching normal M1 chromosomal structure, there were no unusual features detected in *Cap-H2* male sterile metaphase I figures (Figure 3D). Although subtle changes to chromosome morphology would not be detectable, it can be concluded that by metaphase I, gross chromosomal condensation occurs at least somewhat normally in *Cap-H2* strong mutant males. This raises the interesting possibility that a gradual prophase I chromosome condensation is catalyzed by condensin II components in the course of chromosomal territory formation and culminates at M1. Next, a second condensation step to form metaphase I chromosomes occurs, which is only partially dependent or completely independent of condensin II components. Perhaps condensin I or some other factor is the major player for metaphase I chromosome assembly or compensates for condensin II loss.

In contrast to metaphase I, anaphase I is clearly not normal in *Cap-H2* mutants, where instead bridges are often found between segregating sets of chromosomes (Figure 3E, 3F, and 3H). The frequency of these bridges occurs in a manner that matches other phenotypic trends, found in 30.4% of the anaphase I figures for sterile *Cap-H2^Z3-0019^*/*Cap-H2^TH1^* males (n = 102 anaphase I figures), 11.5% for *Cap-H2^Z3-0019^*/*Cap-H2^Z3-5163^* males that are fertile yet undergo 2^nd^ and 3^rd^ chromosome loss (n = 78), and never in the wild-type (n = 90, Figures 3B, 3C, 3G). As with territory formation, Cap-H2 is likely functioning along with Cap-D3 because in two cysts observed from *Cap-D3^EY00456^* homozygous males, 7 of 20 anaphase I figures were bridged (Figure 3I). This anaphase I bridging most likely represents a failure to resolve chromosomal associations prior to segregation as chromatin appears to be stretched between chromosomes moving to opposing poles.

### Anaphase I Bridging in *Cap-H2* Mutants Are Comprised of Homologous and Heterologous Associations

**Figure 4:**
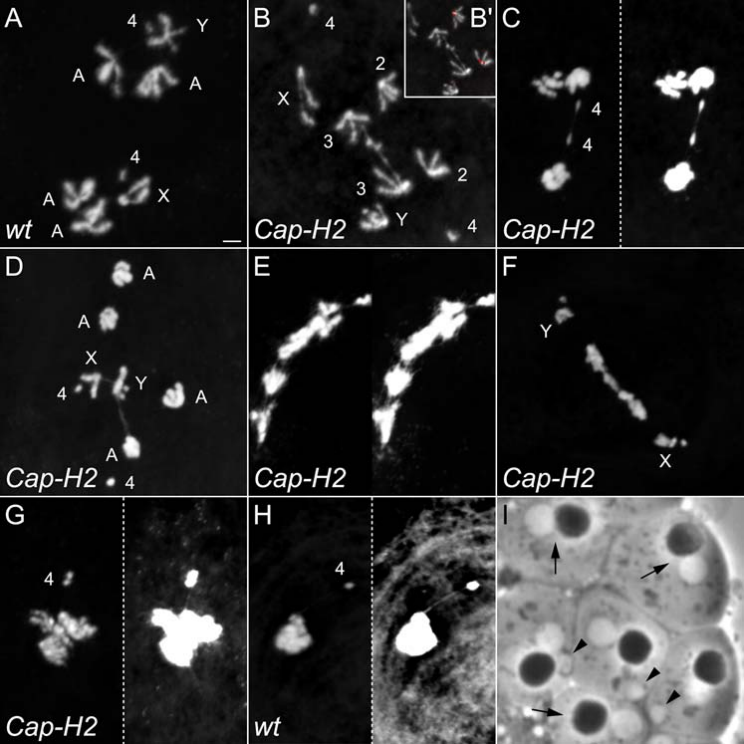
Anaphase I bridges of *Cap-H2* mutant males are comprised of homologous and heterologous chromosomal associations. (A) Wild-type squashed anaphase I. Scale bar indicates 2 µm and serves all anaphase I panels. (B)*Cap-H2^Z3-0019^/Cap-H2^TH1^* mutant where the homologous 3^rd^ chromosomes remain associated while segregating to opposing poles. These were identified as 3^rd^ chromosomes through the use of a 2^nd^ chromosome specific FISH probe that hybridizes to peri-centromeric DNA (B'). (C) *Cap-H2^Z3-0019^/Cap-H2^TH1^* metaphaseI/anaphase I where only the 4^th^ chromosomes have begun to segregate and are connected by a chromatin bridge. Chromosome morphology is more readily observable in the left panel. In the right panel, levels were adjusted to better observe DNA threads between the 4^th^ chromosomes. (D) *Cap-H2^Z3-0019^/Cap-H2^TH1^* anaphase I where a DNA bridge extends from the Y chromosome to a major autosome (2^nd^/3^rd^). A thread between the X and Y chromosomes is also evident and likely represents their site of conjunction called the “collochore” that is commonly observed in wild-type preparations. (E) *Cap-H2^Z3-0019^/Cap-H2^TH1^* anaphase I with extensive DNA bridging. Left panel enables better visualization of chromosome morphology. Levels in the right panel were adjusted to better highlight the extent of bridging. (F) *Cap-H2^Z3-0019^/Cap-H2^TH1^* anaphase I with normal 4^th^ and X/Y segregation, yet major autosomes appear to be involved in complex associations that are generating a chromatin bridge. (G) *Cap-H2^Z3-0019^/Cap-H2^TH1^* prophase/metaphase I where the 4^th^ chromosomes appear to be associated to a heterolog through a DNA thread. Left panel better illustrates chromosome morphology. Levels were adjusted in the right panel to highlight the 4^th^ chromosome-to-heterolog thread. (H) Wild-type prophase/metaphase I where a 4^th^ chromosome appears to be associated to a heterolog through a DNA thread. Left panel better illustrates chromosome morphology. Levels were adjusted in the right panel to highlight the 4^th^ chromosome-to-heterolog thread. (I) *Cap-H2^Z3-0019^/Cap-H2^Z3-0019^* post-meiotic, onion stage cyst cells. Arrows indicate wild-type appearing cells with one dark nebenkern and one light nucleus. Arrow heads highlight cells with micronuclei that may be the manifestation of chromosome loss during anaphase.

To gain further insight into why anaphase I bridges are created in *Cap-H2* and *Cap-D3* mutants, a chromosome squashing technique was employed that enables the visualization of individual anaphase I chromosomes (Figure 4A). With this method, the 4^th^ chromosomes are easily identified because of their dot like appearance. Centromere placement enables the identification of the sex chromosomes, where on the X it is located very near the end of the chromosome (acrocentric) and on the Y is about a quarter of the length from one end (submetacentric). The 2^nd^ and 3^rd^ chromosomes are indistinguishable from one another because of their similar size and placement of the centromere in the middle of the chromosome (metacentric). Whereas bridged anaphase I figures were never observed in wild-type squashed preparations (n = 14; Figure 4A), bridging occurred in 40.5% of those from *Cap-H2^Z3-0019^*/*Cap-H2^TH1^* mutant males (n = 42; Figure 4B–4F).

The chromosome squashing method was utilized to determine the nature of anaphase I bridges, and interestingly, it was concluded that bridging exists between both homologous and heterologous chromosomes (Figure 4). Of the total anaphase I figures from *Cap-H2^Z3-0019^*/*Cap-H2^TH1^* testes, 21.4% appeared to have anaphase I bridging that existed between homologous chromosomes (Figure 4B and 4C). A FISH probe that recognizes 2^nd^ chromosome pericentromeric heterochromatin was used to distinguish 2^nd^ and 3^rd^ chromosomes and demonstrates that linkages in Figure 4B (inset) are between the 3^rd^ chromosomes, perhaps at regions of shared homology. Furthermore, despite not finding 4^th^ chromosome segregation defects in nondisjunction assays (Table 1), the 4^th^ chromosome was bridged in 4.8% of anaphase I figures (Figure 4C). This suggests that chromosome 4 becomes sensitive to further loss of Cap-H2 function in the stronger *Cap-H2^Z3-0019^*/*Cap-H2^TH1^* mutant background.

Persistent associations between homologous chromosomes in anaphase I may be explained by a failure to individualize paired homologs from one another prior to anaphase I entry. It is probable that DNA entanglements normally exist between paired homologous chromosomes as they are likely raveled around one another rather then simply aligned side by side in a linear fashion. Therefore, individualization failure in Cap-H2 mutants may allow entanglements to persist into anaphase I. Cap-H2 may mediate homolog individualization in prophase I, where bivalents do not appear to condense properly in *Cap-H2* mutants (Figure 2). Another plausible scenario is that Cap-H2 functions to antagonize achiasmate homolog conjunction mediated by *teflon*, *MNM*, and *SNM* at some point prior to anaphase I entry.

The other 19% of anaphase I figures that were bridged (n = 42) in the *Cap-H2^Z3-0019^*/*Cap-H2^TH1^* mutant involve heterologous chromosomes (Figure 4D) and cases where bridging is so substantial that its chromosomal nature could not be determined (Figure 4E and 4F). The observed X–Y linkage in Figure 4D is consistent with the XY pairing site, or “collochore,” and occurs in wild-type preparations [45]. The other linkage is an atypical heterologous association occurring between the Y and one of the major autosomes (2^nd^ or 3^rd^). We speculate that the substantially bridged images in Figure 4E and 4F are comprised of associations between heterologous and/or homologous chromosomes. Figure 4F is particularly interesting because the 4^th^ and sex chromosomes appear to have segregated normally, yet the major autosomes remain in an unresolved chromosomal mass. This pattern fits the trend of the nondisjunction studies, where the 2^nd^ and 3^rd^ chromosomes had a heightened sensitivity to *Cap-H2* mutation.

**Figure 5:**
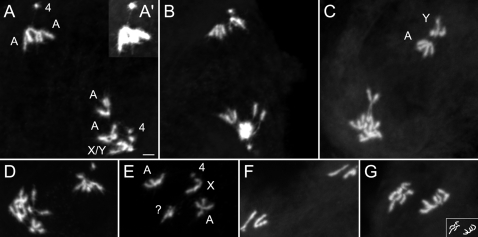
Asymmetric segregation in meiosis I of *Cap-H2* mutants. (A) *Cap-H2^Z3-0019^/Cap-H2^TH1^* anaphase I where one pole appears to contain both X and Y chromosomes. This possibly represents failure in X/Y disjunction and subsequent co-segregation. Also note A', where a 4^th^-to-heterolog thread exists. (B) *Cap-H2^Z3-0019^/Cap-H2^TH1^* anaphase I where one pole contains only two large chromosomes. (C) *Cap-H2^Z3-0019^/Cap-H2^TH1^* anaphase I where one pole contains only one major autosome and the Y chromosome. The other pole appears to carry an extra large chromosome. (D) *Cap-H2^Z3-0019^/Cap-H2^TH1^* anaphase I where one pole contains only two large chromosomes. (E) *Cap-H2^Z3-0019^/Cap-H2^TH1^* prophase I harboring an extra chromosome that may be the result of asymmetric anaphase I segregation. (F) *Cap-H2^Z3-0019^/Cap-H2^TH1^* anaphase II with only one major autosome and the X chromosome. This suggests that an autosome was lost during anaphase I as a consequence of asymmetric segregation. (G) *Cap-H2^Z3-0019^/Cap-H2^TH1^* anaphase II with an extra large chromosome. This may be a consequence of asymmetric segregation in anaphase I. The box at the bottom right illustrates the chromosome configuration.

Because the 4^th^ chromosome naturally tends to be separated from other prometaphase I to anaphase I chromosomes, it was often easily observed to be involved in heterologous chromosomal associations (Figures 4G, 4H, and 5A'). These appear as threads and occurred in 42.5% of metaphase and anaphase I figures (n = 40; Figures 4G and 5A'). Interestingly, 4^th^-to-heterolog threads were also observed in the wild-type, although at a lower frequency of 19% (n = 21, Figure 4H).

Persistent associations between heterologous chromosomes such as that observed in figure 4D and inferred to exist within 4E and 4F may be traced to failed territory formation in *Cap-H2* mutant prophase I. Perhaps interphase chromosomes are naturally entangled with one another and the Cap-H2/Cap-D3 mediated nuclear organization steps that occur during territory formation effectively detangle and individualize them into discrete structures. Alternatively, Cap-H2/Cap-D3 mediated chromosome territory formation may act to prevent the establishment of heterologous entanglements. These are plausible scenarios given that failed territory formation in *Cap-H2/Cap-D3* mutants seemingly leads to persistent intermingling of all chromosomes. Such an environment could provide a likely source of heterologous chromosomal associations. Heterologous associations involving the 4^th^ chromosome may also be entanglements that persist and/or were initiated through failure in territory formation. These cannot however be completely attributed to loss of Cap-H2 function because they were observed in the wild-type (Figure 4H).

The anaphase I bridging in *Cap-H2* mutant males is one likely source for their elevated amount of nullo-2 and nullo-3 sperm (Tables 3 and 4). Chromatin stretched between daughter nuclei may occasionally lead to the creation of sperm lacking whole chromosomes or variable sized chromosomal regions. Bridged anaphase I images in Figure 4 represent likely scenarios where chromosome loss would occur and furthermore, visualization of the post-meiotic “onion stage” from *Cap-H2* mutants is consistent with chromosome loss. With light microscopy, white appearing nuclei within the onion stage are nearly identical in size to the black appearing nebenkern, which represents clustered mitochondria (Figure 4I, arrows). In onion stages from *Cap-H2^Z3-0019^* homozygotes, micronuclei are often observed which may be the manifestation of chromatin lost through anaphase I bridging (Figure 4I, arrowheads).

The associations that create anaphase I bridging between chromosomes moving to opposing poles may also be capable of causing improper cosegregation of homologs. In fact, 9.5% of squashed anaphase I figures (n = 42) are of asymmetrically segregating homologs that were never observed in the wild-type (n = 14). These are consistent with failure in homolog disjunction and subsequent cosegregation to one pole (Figure 5A–5D). These may also be the consequence of associations between heterologous chromosomes that lead to one being dragged to the incorrect pole. As an expected outcome of cosegregation in meiosis I, aneuploidy in prophase II and anaphase II figures was also observed (Figure 5E–5G). Such events likely explain the slight increase in diplo-2 sperm that were heterozygous for the male's 2^nd^ chromosomes (*bw^1^/+* in Table 3). The also provide a likely source for the elevated amount of nullo-2 and nullo-3 sperm (Tables 3 and 4).

**Figure 6:**
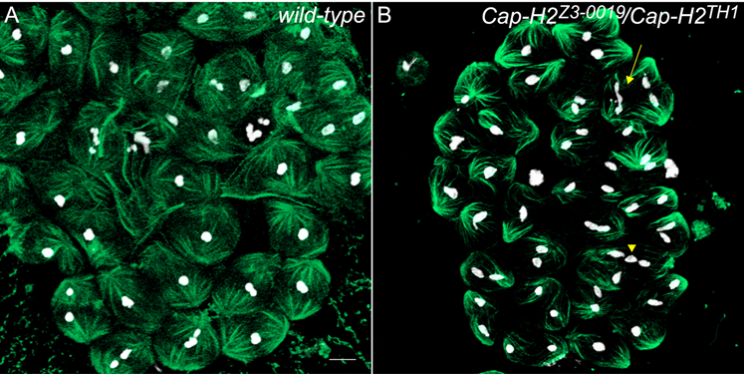
*Cap-H2* mutants are defective in anaphase II segregation. Metaphase II and anaphase II morphologies were compared between wild-type and *Cap-H2^Z3-0019^/Cap-H2^TH1^* mutant males. Testes were stained with DAPI and an anti-tubulin antibody to visualize DNA (white) and microtubules (green), respectively (scale bar in 6A indicates 10 µm). (A) Wild-type metaphase/anaphase II cyst. Metaphase II cells are those where each bivalent has congressed to the metaphase plate and appear as a cluster of DAPI staining material. Anaphase II are those cells with two DAPI staining white clusters, indicating homologous chromosome segregation. (B) Metaphase II and anaphase II figures from a *Cap-H2* strong mutant. Arrow indicates an anaphase II bridge. The arrowhead highlights an anaphase II bridge or lagging chromosome.

While the prevalence of meiotic anaphase I bridging is likely a major contributor to the observed 2^nd^ and 3^rd^ nondisjunction, it cannot be ruled out that the preceding stem cell and gonial mitotic divisions are also defective and lead to aneuploid sperm. This exists as a formal possibility, yet aneuploid meiotic I cells were not observed in squashed *Cap-H2* mutant anaphase I figures where all chromosomes could be distinguished (n = 10). This suggests that pre-meiotic segregation is unaffected. Similarly, anaphase II defects could have contributed to the elevated nullo-2 and nullo-3 sperm and perhaps the slight increase in *bw^1^*/*bw^1^* progeny that would have been generated from meiosis II nondisjunction (Table 3). In fact, anaphase II bridging was observed in 8.7% of *Cap-H2^Z3-0019^*/*Cap-H2^TH1^* anaphase II figures (n = 69, Figure 6), 2.1% of those from *Cap-H2^Z3-0019^*/*Cap-H2^Z3-5163^* males (n = 47), and never in the wild-type (n = 66). Anaphase II defects may occur because of a specific role of Cap-H2 in meiosis II, or alternatively, anaphase II bridging could be attributed to faulty chromosome assembly or individualization in meiosis I.

### Teflon Mutations Suppress Cap-H2 Mutant Anaphase I Bridging Defects

**Figure 7:**
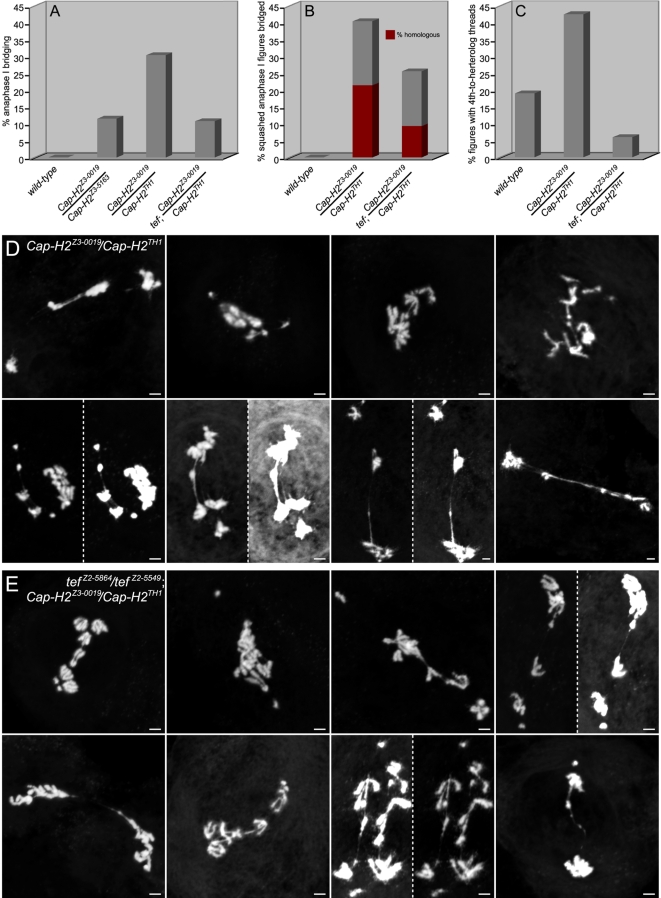
*Teflon* mutations rescue the homologous and heterologous chromosomal associations of *Cap-H2* mutants. (A) Chromatin bridges were not observed in wild-type anaphase I figures (n = 90), yet occur in *Cap-H2* mutants in a manner directly related to allelic strength. Bridges occurred 11.5% of anaphase I figures from fertile *Cap-H2^Z3-0019^/Cap-H2^Z3-5163^* males (n = 78) and in 30.4% of those from sterile *Cap-H2^Z3-0019^/Cap-H2^TH1^* males (n = 102). Mutations in *eflon* suppress the anaphase I bridging of sterile *Cap-H2* mutant males to 10.8% (n = 74, *p*<1×10^−6^, X^2^ test). (B) Chromatin bridges were not observed in squashed anaphase I figures from wild-type preparations (n = 14), yet occurred in 40.5% of anaphase I figures from *Cap-H2^Z3-0019^/Cap-H2^TH1^* males (n = 42). Bridges where the chromosomal nature could not be determined constitute 19% of the anaphase I figures (as indicated by the gray portion of the bar graph). The remaining 21.4% appeared to be bridging that existed between homologous chromosomes (burgundy portion). Mutations in *eflon* suppressed overall anaphase I bridging in squashed preparations to 25.6% (n = 43, *p*<0.05, X^2^ test) and homologous chromosome bridging to 9.3% (*p*<0.1, X^2^ test). (C) *Cap-H2^Z3-0019^/Cap-H2^TH1^* males have an elevated amount of meiosis I 4^th^-to-heterolog threads relative to wild-type and these are suppressible by *teflon* mutations. In prometaphase I to telophase I, 4^th^-to-heterolog threads occur in 19% of wild-type (n = 21), 42.5% in *Cap-H2^Z3-0019^/Cap-H2^TH1^* (n = 40), and 6% in *tef^Z2-5549^*/*tef^Z2-5864^*; *Cap-H2^Z3-0019^*/*Cap-H2^TH1^* (n = 50) squashed figures. The ability of *teflon* mutations to rescue *Cap-H2* mutant 4^th^-to-heterolog threads was significant (*p*<0.00001, X^2^ test). (D) Bridged *Cap-H2^Z3-0019^/Cap-H2^TH1^* anaphase I squashed figures. See also Figures 4 and 5. (A) Bridged *tef^Z2-5864^/tef^Z2-5549^*; *Cap-H2^Z3-0019^/Cap-H2^TH1^* squashed anaphase I figures.

The protein Teflon is implicated in the maintenance of *Drosophila* male meiosis I autosome conjunction as *teflon* mutants lose autosomal associations prior to anaphase I [31]. To investigate whether persistent associations between homologous chromosomes in anaphase I of *Cap-H2* mutants (Figure 4B and 4C) are Teflon dependent, *teflon* mutations were crossed into a *Cap-H2* mutant background and the frequency of anaphase I bridging was assessed. While 30.4% of anaphase I figures from *Cap-H2^Z3-0019^*/*Cap-H2^TH1^* males were bridged (n = 102), bridging existed within only 10.8% of anaphase I figures from *tef^Z2-5549^*/*tef^Z2-5864^*; *Cap-H2^Z3-0019^*/*Cap-H2^TH1^* males (n = 74, *p*<1×10^−6^, X^2^) (Figure 7A). Furthermore, in squashed preparations anaphase I bridging was decreased from 40.5% in *Cap-H2^Z3-0019^*/*Cap-H2^TH1^* males (n = 42) to 25.6% in the *tef^Z2-5549^*/*tef^Z2-5864^*; *Cap-H2^Z3-0019^*/*Cap-H2^TH1^* double mutants (n = 43, *p*<0.05, X^2^).

The ability of *teflon* mutations to rescue *Cap-H2* mutant anaphase I bridging suggests that Cap-H2 functions to antagonize Teflon mediated autosome conjunction. This may entail deactivation of an achiasmate conjunction complex consisting of MNM, SNM, and perhaps Teflon, at some point prior to the metaphase I to anaphase I transition. Consistent with this hypothesis, the percent of anaphase I figures where homologous chromosomes appeared to be bridged were decreased from 21.4% in the *Cap-H2^Z3-0019^*/*Cap-H2^TH1^* mutants (n = 42) to 9.3% in *tef^Z2-5549^*/*tef^Z2-5864^*; *Cap-H2^Z3-0019^*/*Cap-H2^TH1^* males (n = 43, *p*<0.1, X^2^, Figure 7B).

As an important alternative to Cap-H2 functioning to antagonize an achiasmate homolog conjunction complex, it may be that wild-type Teflon exacerbates DNA associations between chromosomes. For example, perhaps Teflon linked homologs are now particularly prone to becoming entangled. Under this scenario, *teflon* mutations may decrease the opportunity for DNA entanglements to be introduced between homologs because of their spatial distancing from one another during late prophase I to metaphase I. Given the formal possibility of both models, we conclude that Cap-H2 functions to either remove *teflon* dependent conjunction and/or to resolve chromosomal entanglements between homologs.

The remaining bridged anaphase I figures from squashed preparations in *tef^Z2-5549^*/*tef^Z2-5864^*; *Cap-H2^Z3-0019^*/*Cap-H2^TH1^* males were uninterpretable making it impossible to assess whether *Cap-H2* mutant heterologous anaphase I bridging was also rescued by *teflon* mutation. However, 4^th^-to-heterolog threads were greatly suppressed by *teflon* mutations, decreasing from 42.5% (n = 40) to only 6% (n = 50, *p*<0.00001, Figure 7C). This is a surprising result given that Teflon has been described as a mediator of associations between homologous chromosomes. One plausible explanation is that Teflon can exacerbate heterologous chromosomal associations. This may occur when Teflon establishes autosomal conjunction in a prophase I nucleus where territory formation had failed. Cap-H2 may also antagonize a Teflon mediated autosomal conjunction complex that might mistakenly establish conjunction between heterologs when territories do not form.

As described above, completely male sterile *Cap-D3* and *Cap-H2* allelic combinations exist and *Cap-H2* mutant males lack mature sperm in their seminal vesicles (Figure 1A and 1B). One possible explanation for this result is that chromosome damage created during anaphase bridging in the *Cap-H2* mutants causes spermatogenesis to abort. This scenario seems less likely because *tef^Z2-5549^*/*tef^Z2-5864^* rescued *Cap-H2^Z3-0019^*/*Cap-H2^TH1^* anaphase I bridging to levels near that of fertile *Cap-H2* mutants, yet *tef^Z2-5549^*/*tef^Z2-5864^*; *Cap-H2^Z3-0019^*/*Cap-H2^TH1^* males were still found to be completely sterile. This points toward another function for Cap-H2 in post-meiotic steps of spermatogenesis.

### Cap-H2 and Cap-D3 Function to Resolve Chromosomal Associations to Enable Meiosis I Segregation

**Figure 8:**
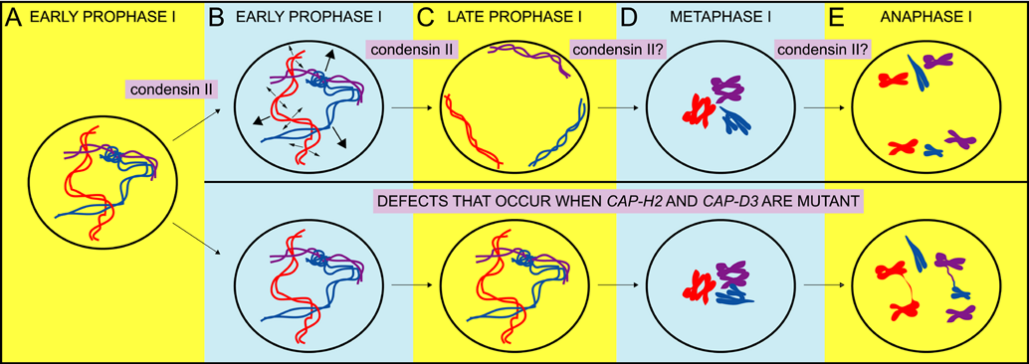
A working model for the function of condensin II in the resolution of DNA entanglements between homologous and heterologous chromosomes prior to *Drosophila* male meiotic anaphase I. (A) In interphase nuclei, heterologous chromosomes intermingle and can naturally become entangled with one another because of their threadlike structure. Homologous chromosomes are also entangled as the pairing process likely leads to their raveling around one another. (B) In early prophase I, condensing II functions to promote territory formation. This either entails partial condensation of each bivalent into compact clusters of chromatin and/or the active sequestration of each bivalent into discrete chromosomal territories. In the course of condensing II mediated territory formation, DNA entanglements between heterologous chromosomes are resolved and/or their introduction is prevented (both depicted by large arrows). Paired homologs may also be individualized from one another during condensing II mediated chromosome organizational steps that occur during prophase I (depicted by small arrows). (B) Condensin II successfully promotes territory formation by late prophase I and heterologous chromosomes are individualized from one another. It is unclear whether condensin II functions after territory formation to further individualize homologs. In condensin II mutants, failure in the chromosome organizational steps during territory formation leads to entanglement persistence. (C) In wild-type metaphase I, little to no entanglements between chromosomes still exist. It is unclear, but feasible that condensin II mediated individualization is still present during metaphase I or at the transition into anaphase I. When condensin II is not functional, entanglements between homologous and heterologous chromosomes still exist. (D) In wild-type anaphase I, homologous chromosomes segregate from one another to daughter cells. When condensin II is mutant, persistent entanglements between heterologous and homologous chromosomes that were not resolved prior to anaphase I become revealed as chromatin bridges. Persistent entanglements may also lead to improper cosegregation of homologs or heterolog dragging to one pole (not shown). Note that our data cannot distinguish between the two following possible mechanisms: Condensin II resolves DNA entanglements prior to anaphase I and/or condensin II antagonizes another type of chromosomal association. In fact, mutations in *teflon*, a factor that promotes male autosomal conjunction, were capable of rescuing anaphase I bridging in *Cap-H2* mutants. While this may be because Teflon protein induces and/or exacerbates DNA entanglements between heterologous and homologous chromosomes, it is plausible that Cap-H2 can antagonize a Teflon mediated homolog conjunction complex.

Figure 8 illustrates a working model of condensin II in *Drosophila* male meiosis to resolve both heterologous and homologous chromosomal associations. We speculate that these associations likely consist of DNA entanglements that naturally become introduced between interphase chromosomes due to their threadlike nature (Figure 8A). The studies herein identified a function for condensin II during prophase I, when paired homologous chromosomes become partitioned into discrete chromosomal territories [33]–[36]. We propose that condensin II either promotes this partitioning, by actively sequestering bivalents into different regions of the nucleus, or functions to perform prophase I chromosome condensation. It is important to stress that in both scenarios, the role of condensin II mediated territory formation is to ensure the individualization of heterologous chromosomes from one another (Figure 8B, large arrows). When sequestration into territories and/or condensation of the bivalents do not take place, i.e. in the condensin II mutants, individualization does not occur, heterologous entanglements persist into anaphase I, and chromosomes may become stretched to the point where variable sized chromosomal portions become lost (Figure 8E). Persistent heterologous entanglements may also lead to one chromosome dragging another to the incorrect pole (not shown).

Despite what appears to be failed chromosome condensation in prophase I of *Cap-H2* mutants, by metaphase and anaphase I no obvious defects in chromosome condensation were observed (Figures 3, 4, 5, and 7). This suggests that sufficient functional *Cap-H2* is present in this mutant background to promote metaphase/anaphase I chromosome condensation. Alternatively, perhaps another factor fulfills this role and/or compensates for condensin II loss. This parallels *Cap-G* mutants, where embryonic mitotic prophase/prometaphase condensation was abnormal, yet metaphase figures appeared wild-type [38]. In *Drosophila*, mutant and RNAi knockdown studies of condensin complex subunits in mitosis lead to a range of phenotypes, from complete failure in condensation [46] to seemingly normal axial shortening, but failure in chromatid resolution [37],[39]. The variable phenotypes produced from these studies may reflect differences in cell type specific demand for condensin subunit dosage/activity.

Anaphase I figures of *Cap-H2* mutants also revealed persistent entanglements between homologous chromosomes that may be at regions of shared homology. We suggest that the paired state of homologs initiates or introduces the opportunity for DNA entangling between homologs and that condensin II functions to resolve these prior to segregation. A likely scenario is that this occurs during prophase I, where chromosome condensation appears abnormal in *Cap-H2* and *Cap-D3* mutants. Perhaps condensin II mediated prophase I condensation functions to individualize intertwined homologous chromosomes prior to segregation (Figure 8B, small arrows). It is also plausible that condensin II homolog individualization continues up until anaphase I.

We have found that mutations in *teflon*, a gene required for autosomal pairing maintenance, were capable of suppressing anaphase I bridging in *Cap-H2* mutant males. Specifically, both homologous and heterologous chromosomal bridging were decreased in the *teflon/Cap-H2* double mutant. This may occur because Teflon is capable of exacerbating DNA entanglements, if for example persistent homolog conjunction provides more opportunity for entanglements between homologs to be introduced. Teflon may also exacerbate entanglements between heterologous chromosomes. This might be especially true in a Cap-H2 mutant background with failed territory formation, as Teflon mediated autosomal conjunction may augment the extent of entangling.

It is also plausible that Cap-H2 acts as an antagonist of Teflon mediated autosomal conjunction. Perhaps autosomal homologous associations persist into anaphase I of *Cap-H2* mutants because a homolog conjunction complex was not disabled prior to the metaphase I to anaphase I transition. However, Cap-H2 as an antagonist of Teflon cannot explain persistent heterologous associations into anaphase I, unless Teflon is capable of mistakenly introducing conjunction between heterologous chromosomes. The opportunity for this might exist in a *Cap-H2* mutant prophase I nucleus where heterologs continue to intermingle because of failed territory formation.

An interesting result in our course of studies was the heightened amount of chromosome 2 and 3 nondisjunction in weaker male fertile Cap-H2 allelic combinations, whereas the sex and 4^th^ chromosomes were unaffected. This is reminiscent of mutants from several other genetic screens that only affected the segregation of specific chromosomes or subsets [32], [47]–[51]. However, given that sex and 4^th^ chromosome segregation defects were observed in the stronger male sterile Cap-H2 mutant background, we propose that condensin II functions upon all chromosomes, yet the 2^nd^ and 3^rd^ require the greatest functional Cap-H2 dose for their proper segregation. This sensitivity of the 2^nd^ and 3^rd^ chromosomes may be due to their greater total amount of DNA utilized in homolog pairing and pairing maintenance activities. For example, perhaps longer stretches of paired DNA are more prone to entanglements or require more achiasmate conjunction factors and therefore necessitate higher levels of Cap-H2 individualization or disengagement activity. As an interesting corollary to support this theory, weak *teflon* mutations only lead to 4^th^ chromosome missegregation, while the other autosomes segregate normally [31]. This suggests that the 4^th^ chromosomes are more sensitive to Teflon dosage because of their fewer sites of conjunction.

The majority of the data provided in this manuscript were on our studies of mutant *Cap-H2* alleles, however, we found that a homozygous viable *Cap-D3* mutant also failed to form normal chromosomal territories and exhibited anaphase I chromosome bridging. This provides support that these two proteins are functioning together within a condensin II complex. It is important to point out however, that to date there is no data in *Drosophila* to support that these proteins physically associate with each other or with other condensin subunits, namely SMC2 and SMC4 (a *Drosophila Cap-G2* has yet to be identified with computational attempts) [8].

At this point in our studies of putative condensin II subunits in disjunction of achiasmate male homologous chromosomes, we cannot distinguish between possible scenarios that Cap-H2 and Cap-D3 act to disentangle chromosomes through individualization activity, that they function as antagonists of Teflon dependent achiasmate associations, or a combination of both activities. The fact that Teflon mutations do rescue Cap-H2 anaphase I bridging defects is an especially intriguing result as it points toward a molecular mechanism for Cap-H2 as an antagonist of achiasmate associations. While three genes have been found to promote achiasmate conjunction (*teflon*, *MNM*, and *SNM*), no factors have been identified that act to negatively regulate conjunction and allow homologs to disengage at the time of segregation. Interestingly, one conjunction factor, SNM, is orthologous to the cohesin subunit Scc3/SA that appears to be specialized to engage achiasmate homologs [30]. Condensin has been shown to antagonize cohesins in budding yeast meiosis [52] and mitotic human tissue culture cells [53]. This raises the possibility that a conserved molecular mechanism exists for condensin II as a negative regulator of SNM in *Drosophila* male meiosis. The investigation of Teflon, MNM, and SNM protein dynamics in a *Cap-H2* mutant background will be an important set of future studies to help decipher the function of Cap-H2 in achiasmate segregation mechanisms.

Homologous chromosomal individualization in meiosis I has been previously documented as a condensin complex catalyzed activity in *C. elegans* as homologs remained associated in *hcp-6/Cap-D3* mutants even in the absence of recombination and sister chromatid cohesion [6]. Here we demonstrated that condensin subunits are also required to individualize heterologous chromosomes from one another prior to anaphase I. As discussed above, this is likely through condensin II mediated chromosome organizational steps that occur during prophase I territory formation. This suggests that *Drosophila* males carry out territory formation to disfavor associations between heterologs, while also enriching for interactions between homologs. This model is particularly interesting as it may point toward an adaptation of *Drosophila* males to ensure meiotic I segregation in a system lacking a synaptonemal complex and recombination.

## Materials And Methods

### Cytology and Immunofluorescence

To visualize sperm head (DAPI) and tail (*don juan-GFP*) content in the seminal vesicles, males were restricted from females for ten days, then testes were dissected and fixed as previously described for whole mounted ovaries [54]. Meiotic microtubules were detected with rat anti-alpha tubulin antibodies (Serotec, MCA78G and MCA77G) at 1∶40 each and a FITC-conjugated donkey anti-rat secondary (Jackson ImmunoResearch, #112-095-167) at 1∶200. Immunofluorescence was conducted following protocols 5.2 and 5.6 from ref [55], with the addition of two extra final PBS washes, the second to last containing 100 ng/ul DAPI. DAPI stained chromosome squashes were prepared as detailed in protocol 1.9, method #3 w/o steps necessary for immuno-detection from ref. [56]. Testes were opened to release cells while in fixative on a siliconized coverslip prior to lowering a non-siliconized slide and squashing. Subsequent FISH to anaphase chromosome spreads was conducted as detailed in protocol 2.9 in ref. [57]. An (AACAC)_6_ oligonucleotide end labeled with terminal deoxytransferase (Roche 03333566001) and reagents provided in the ARES Alexa Fluor 546 DNA labeling kit (Invitrogen A21667) were utilized to fluorescently detect 2^nd^ chromosome pericentromeric heterochromatin. All imaging was performed with a Zeiss Laser Scanning Microscope, LSM 510 Meta, and the acquisition software LSM 510 Meta, version 4.0. Images in figure 1 were captured with a Plan-Apochromat 20×/0.8 objective at an image bit depth of 8 bit. All other images were acquired with a Plan-Apochromat 63×/1.4 Oil DIC objective at an image bit depth of 8 bit. Appropriate filters and dichroic mirrors for fluorochromes DAPI, Alexa Fluor 546, and FITC were used where applicable.

### Male Fertility Time Course and Fertility Tests

To test for male fertility, 10 mutant males were crossed to 20 wild-type (Oregon R) virgin females and monitored frequently for the presence of larvae. To score fertility over time of the *Cap-H2 trans*-heterozygous and heterozygous control males, 10, 1–4 day old males were placed with 30, 1–5 day old virgin females in containers with grape juice agar plates and wet yeast. Flies were transferred to new plates every 24 hours for 4 days, but on the 4^th^, 8^th^, and 12^th^ days, only males were kept and placed with a new batch of 1–5 day old virgin females. This scheme was carried out over a period of 16 days and in triplicate. For the *SMC4*; *Cap-H2* double mutant studies, the strategy is as detailed above, except only 20 virgin females were used for each brood. To score hatch rates, the percent of eggs that hatched (n = 200 total eggs/plate) was scored from randomly selected regions of each plate 48 hours after parents were removed.

### 4^th^ Chromosome Nondisjunction Tests

Five *Cap-H2^Z3-0019^/Cap-H2^Z3-5163^*; *spa^pol^/+* males were crossed to fifteen *C(4)EN*, *ci ey* females at 25°C on standard fly food. As controls, the same experimental design was carried out with *Cap-H2^Z3-0019^*/*TM6B*, *Hu*; *spa^pol^*/+ or *Cap-H2^Z3-5163^*/*TM6B*, *Hu*; *spa^pol^*/+ males. Males and virgin females were 2–3 days old and the experimental cross was done in replicate, while the controls were only performed once. Parents were twice flipped into a new bottle after three days and then discarded from their final bottle after three days. Progeny were scored on the 13^th^, 15^th^, and 18^th^ day after parents were placed into the bottle. Because the 4^th^ chromosome in these females is attached, they produce eggs that either carry the compound *C(4)EN*, *ci ey* chromosome (diplo-4) or no 4^th^ chromosome (nullo-4). The fertilization of nullo-4 eggs by normal haploid sperm creates nullo-4/+ and nullo-4/*spa^pol^* progeny. Both of these will develop into very small flies (Minute) from only carrying one 4^th^ chromosome, with the latter also *spa^pol^*. When normal haplo-4 sperm fertilize *C(4)EN*, *ci ey*/0 eggs, *C(4)EN*, *ci ey*/+ or *C(4)EN*, *ci ey*/*spa^pol^* progeny are produced. These both appear wild-type from the wild-type alleles of *ci* and *ey* on the paternal 4^th^ and wild-type *spa^pol^* on the *C(4)EN* chromosome. There are two exceptional classes from male chromosome missegregation events that are detectable with this assay. The first is when nullo-4 sperm fertilize *C(4)EN*, *ci ey*/0 eggs to produce *ci ey* offspring. The second are sperm diplo-4 and homozygous for *spa^pol^* fertilizing nullo-4 eggs to create *spa^pol^* offspring. The following exceptional classes go undetected with this assay because they are phenotypically wild-type: +/+, *spa^pol^/+*, *spa^pol^/spa^pol^* sperm that fertilize *C(4)EN* eggs or +/+, *spa^pol^/+* and all triplo-4 and tetra-4 sperm possibilities that fertilize nullo-4 eggs. Therefore, the % 4^th^ chromosome nondisjunction is likely an underestimate. This assay was adapted from that described in ref. [32].

### Sex Chromosome Nondisjunction Tests

Ten males, that were 2–3 days old, were crossed to 17 virgin females that were 0–3 days old at 25°C. Males each carried a Y chromosome with an X translocation containing the wild-type *yellow* gene. Females carried an attached X chromosome: *C(1)RM*, *y^2^ su(wa)wa*. In this assay, the viable offspring from sperm bearing the normal sex chromosome content, either one X or one Y, will be *y^1^w^1^*/*nullo-X* (*yw*, XO male) or *y+Y*/*C(1)RM*, *y^2^ su(w^a^)w^a^* (*y+*, XXY female) (nullo-X/Y and triplo-X are lethal combinations). If exceptional classes of sperm are created that are diplo-X, XY, XXY, or lack either sex chromosome entirely (nullo-X or nullo-Y), then *yellow white* females, *white* males, *white* females, or *yellow* females will be produced, respectively. With this assay it cannot be determined whether offspring carry an extra Y chromosome. This experiment is adapted from that detailed in ref. [40].

### 2^nd^ Chromosome Nondisjunction Tests

The line *C(2)EN*, *b pr* carries second chromosomes that are fused, referred to as “compound” chromosomes, that segregate together as a unit and therefore gametes are created that are either nullo-2 or diplo-2. Because any chromosome 2 content other than diplo-2 is lethal, viable offspring only occur from the fertilization of nullo-2 eggs by diplo-2 sperm or diplo-2 eggs by nullo-2 sperm. Therefore, if any offspring are created when crossing males to *C(2)EN*, *b pr* virgin females, then chromosome mis-segregation had occurred in the generation of male gametes. The males used in this experiment were heterozygous for a mutant allele of *brown* (*bw^1^*) that is an insertion of a 412 retrotransposable element into the *brown* gene. In this assay, there are four classes of sperm that can successfully fertilize eggs from *C(2)EN* bearing females that can then develop into adult flies: nullo-2, diplo-2 (*bw^1^/bw^1^*), diplo-2 (*bw^1^/+*), and diplo-2 (+/+). Progeny from nullo-2 sperm fertilizing diplo-2 eggs have the *b pr* phenotype. Those from *bw^1^/bw^1^* sperm fertilizing nullo-2 eggs have the *bw* phenotype. Progeny from *bw^1^/+* and +/+ sperm fertilizing nullo-2 eggs both appear wild-type. To distinguish between these two wild-type phenotypic classes, a PCR test was developed that could detect the presence of the *bw^1^* mutant allele by utilizing the 412 element insertion in the *brown* gene. Thus, with forward primer tattatctgagtgagttttctcgag that anneals to the 412 element and reverse primer ttcacccacatcatcctcat that anneals to the *brown* gene, a 874 bp PCR product is generated only from *bw^1^/+* and never from +/+ flies. Furthermore, with forward primer ggtgatctgcaattagggat and the same reverse primer as above (ttcacccacatcatcctcat), an ∼571 bp fragment amplifies from the wild-type *brown* locus within both *bw^1^/+* and +/+ flies, and serves as a positive control. Wild-type in these assays was the parental line from the *Z3-0019* and *Z3-5163* backgrounds [58] crossed to Oregon R (*bw^1^/+*; *st^1^*/+). Similarly, *Cap-H2* heterozygous males were generated from a cross to Oregon R. Ten 1–3 day old males were crossed to twenty 1–5 day old virgin *C(2)EN*, *b pr* females at 25°C. This was replicated 19 times for the *bw^1^/+*; *Cap-H2^Z3-0019^/Cap-H2^Z3-5163^* males, 12 for *bw^1^/+*; *Cap-H2^Z3-0019^/+*, 20 for *bw^1^/+*; *Cap-H2^Z3-5163^*/+, and 15 for *bw^1^/+*; *st^1^*/+. The parents were kept in the original vial for a total of 5 days, flipped to a new vial for 5 more days, and then discarded. The progeny were scored on the 13^th^, 15^th^, and 18^th^ day after parents were placed together into a vial.

### 3^rd^ Chromosome Nondisjunction Tests

Like the second chromosome, any chromosome 3 content other than diplo-2 is lethal, so viable offspring only occur from the fertilization of nullo-3 eggs by diplo-3 sperm or diplo-3 eggs by nullo-3 sperm. This experiment was therefore set up in the same way as the 2^nd^ chromosome nondisjunction tests, except that *C(3)EN*, *st cu e* females were used, three replicates were performed, parents were kept in vials for 3 days and flipped twice, and these crosses were done at room temperature (21–23°C). In this assay, there are four classes of sperm that can successfully fertilize eggs from *C(3)EN* bearing females that can then develop into adult flies: nullo-3, diplo-3 (heterozygous for paternal 3^rd^ chromosomes), diplo-3 (homozygous for one of the paternal 3^rd^ chromosomes) and diplo-3 (homozygous for the other paternal 3^rd^ chromosome). The *Cap-H2^Z3-0019^* chromosome is marked with *ru*, *h*, *st*, *sr*, *e*, and *ca*, while the *Cap-H2^Z3-5163^* chromosome is marked with only *st*. Using Cap-*H2^Z3-0019^/Cap-H2^Z3-5163^* males as an example, the following describes how nullo-3 and the three different diplo-3 progeny classes were distinguished. Progeny from nullo-3 sperm fertilizing *C(3)EN*, *st cu e*, eggs have the *st cu e* phenotype. Those from diplo-3, *Cap-H2^Z3-0019^/Cap-H2^Z3-0019^*, sperm fertilizing nullo-3 eggs would be *ru h st sr e ca*. The progeny from diplo-3, *Cap-H2^Z3-0019^/Cap-H2^Z3-5163^* and *Cap-H2^Z3-5163^/Cap-H2^Z3-5163^*, sperm fertilizing nullo-3 eggs both develop into *st* animals. These were distinguished by crossing to *ru h st Cap-H2^Z3-0019^ st e ca/TM6B, Hu Tb e ca* flies and scoring F2 progeny.

### Scoring of Squashed Preparations for Anaphase I Bridging and 4^th^ Chromosome-to-Heterolog Threads

The percentage of bridged anaphase I figures where chromosomes are oriented such that their identity is unambiguous is low. Additionally, anaphase I chromosomes quickly decondense upon entry into telophase I, reducing the overall frequency of anaphase I figures where chromosomes can be observed. Thus, the stronger *Cap-H2^Z3-0019^*/*Cap-H2^TH1^* allelic combination was analyzed to increase the likelihood of visualizing interpretable bridged figures. Bridges were scored as homologous when they appeared to connect morphologically similar chromosomes, based on size and centromere location (see text) that appeared to be segregating away from one. It was concluded that the 4^th^ chromosome was involved in a heterologous association during meiosis I when a DAPI staining thread extended to another non-4^th^ chromosome. Thus, images were only scored when this thread clearly was connected to a heterolog, or the other 4^th^ was present and it was clear that it did not participate in the thread. In the wild-type figures where 4^th^ chromosome threads were observed, it could not be concluded whether the thread extended to another 4^th^ or a heterolog. The data in figure 7C for the wild-type may therefore be an overestimate of 4^th^-to-heterolog threads because threads may actually connect homologs.

### Fly Stocks

*bw*; *st Z3-0019*/*TM6B*, *Hu Tb e ca* and *bw*; *st Z3-5163*/*TM6B*, *Hu Tb e ca* were obtained from Charles Zuker [58] and were identified in a previously detailed genetic screen [59]. A recombinant chromosome of the *Z3-0019* line, *ru h st Cap-H2^Z3-0019^ sr e ca/TM6B*, *Hu Tb e ca* was used for all experiments herein. The *Cap-H2^TH1^* allele was found on the *Df(3L)W10* bearing chromosome during the course of complementation studies that will be described elsewhere. The deficiency *Df(3L)W10* was recombined away from the *Cap-H2^TH1^* bearing chromosome and instead *ru h st Cap-H2^TH1^ Sb*[*sbd-2*]/*TM6B*, *Hu Tb e ca* was utilized in these studies. The stocks *SMC4^k08819^*, *spa^pol^*, *C(2)EN*, *b pr*, *C(3)EN*, *st cu e*, *Df(3R)Exel6159*, *Cap-D3^EY00456^*, and *Df(2L)Exel7023* were obtained from the Bloomington stock center. John Tomkiel provided the following stocks: *cn tef^Z2-5549^ bw*/*CyO*, *cn tef^Z2-5864^ bw*/*CyO*, and *y w sn*; *C(4)EN*, *ci ey*. The *don juan-GFP*/*CyO* and *C(1)RM*, *y^2^ su(w^a^)w^a^* were received from Terry Orr-Weaver.

## Supporting Information

Figure S1 *Cap-H2* and *Cap-D3* denoting locations of each mutant allele. Coding regions are depicted in black and 5′ and 3′ UTRs in gray. (A) *Cap-H2* genomic locus showing splicing patterns found in a *Cap-H2* cDNA library. *Cap-H2^TH1^* is a GT to GC alteration in the first intron's splice acceptor site (tgaagaagcggaagcgggt to tgaagaagcggaagcgggc) and was found on the chromosome carrying *Df(3L)W10*. *Cap-H2^Z3-0019^* carries two SNPs. The first (SNP#1) is an A to T base change in the first intron (gaagcgggtaagcatcca to gaagcgggtaagcatcct) and the second (SNP#2) a G to A mutation changing tagatccgggactgg into tagatccgggactag that switches a tryptophan codon into a stop codon. *Cap-H2^Z3-5163^* is an aberration that has only been defined as to the right of a PstI restriction site ( ctgcagatcctcaaatac) and to the left of a forward primer binding site gttaatggacgatagggcacgtt (as characterized with preliminary southern and PCR analyses) and is consistent with either an insertion or rearrangement. (B) *Cap-D3* genomic locus as detailed in the *Drosophila melanogaster* genome release 4.3. Allele *Cap-D3^EY00456^* is a P-element insertion into the third exon.
(6.6 MB TIFF)

## References

[1] Hassold T, Hunt P (2001). To err (meiotically) is human: the genesis of human aneuploidy.. Nat Rev Genet.

[2] Swedlow JR, Hirano T (2003). The making of the mitotic chromosome: modern insights into classical questions.. Mol Cell.

[3] Wang JC (2002). Cellular roles of DNA topoisomerases: a molecular perspective.. Nat Rev Mol Cell Biol.

[4] Uemura T, Ohkura H, Adachi Y, Morino K, Shiozaki K (1987). DNA topoisomerase II is required for condensation and separation of mitotic chromosomes in S. pombe.. Cell.

[5] Gimenez-Abian JF, Clarke DJ, Devlin J, Gimenez-Abian MI, De la Torre C (2000). Premitotic chromosome individualization in mammalian cells depends on topoisomerase II activity.. Chromosoma.

[6] Chan RC, Severson AF, Meyer BJ (2004). Condensin restructures chromosomes in preparation for meiotic divisions.. J Cell Biol.

[7] Hirano T (2005). Condensins: organizing and segregating the genome.. Curr Biol.

[8] Ono T, Losada A, Hirano M, Myers MP, Neuwald AF (2003). Differential contributions of condensin I and condensin II to mitotic chromosome architecture in vertebrate cells.. Cell.

[9] Yeong FM, Hombauer H, Wendt KS, Hirota T, Mudrak I (2003). Identification of a subunit of a novel Kleisin-beta/SMC complex as a potential substrate of protein phosphatase 2A.. Curr Biol.

[10] Hagstrom KA, Holmes VF, Cozzarelli NR, Meyer BJ (2002). C. elegans condensin promotes mitotic chromosome architecture, centromere organization, and sister chromatid segregation during mitosis and meiosis.. Genes Dev.

[11] Kimura K, Hirano T (1997). ATP-dependent positive supercoiling of DNA by 13S condensin: a biochemical implication for chromosome condensation.. Cell.

[12] Kimura K, Rybenkov VV, Crisona NJ, Hirano T, Cozzarelli NR (1999). 13S condensin actively reconfigures DNA by introducing global positive writhe: implications for chromosome condensation.. Cell.

[13] Hirano T (2006). At the heart of the chromosome: SMC proteins in action.. Nat Rev Mol Cell Biol.

[14] Geiman TM, Sankpal UT, Robertson AK, Chen Y, Mazumdar M (2004). Isolation and characterization of a novel DNA methyltransferase complex linking DNMT3B with components of the mitotic chromosome condensation machinery.. Nucleic Acids Res.

[15] Lupo R, Breiling A, Bianchi ME, Orlando V (2001). Drosophila chromosome condensation proteins Topoisomerase II and Barren colocalize with Polycomb and maintain Fab-7 PRE silencing.. Mol Cell.

[16] Machin F, Paschos K, Jarmuz A, Torres-Rosell J, Pade C (2004). Condensin regulates rDNA silencing by modulating nucleolar Sir2p.. Curr Biol.

[17] Xu Y, Leung CG, Lee DC, Kennedy BK, Crispino JD (2006). MTB, the murine homolog of condensin II subunit CAP-G2, represses transcription and promotes erythroid cell differentiation.. Leukemia.

[18] Sullivan M, Higuchi T, Katis VL, Uhlmann F (2004). Cdc14 phosphatase induces rDNA condensation and resolves cohesin-independent cohesion during budding yeast anaphase.. Cell.

[19] Bhat MA, Philp AV, Glover DM, Bellen HJ (1996). Chromatid segregation at anaphase requires the barren product, a novel chromosome-associated protein that interacts with Topoisomerase II.. Cell.

[20] Coelho PA, Queiroz-Machado J, Sunkel CE (2003). Condensin-dependent localisation of topoisomerase II to an axial chromosomal structure is required for sister chromatid resolution during mitosis.. J Cell Sci.

[21] Bhalla N, Biggins S, Murray AW (2002). Mutation of YCS4, a budding yeast condensin subunit, affects mitotic and nonmitotic chromosome behavior.. Mol Biol Cell.

[22] D'Ambrosio C, Kelly G, Shirahige K, Uhlmann F (2008). Condensin-dependent rDNA decatenation introduces a temporal pattern to chromosome segregation.. Curr Biol.

[23] Li X, Nicklas RB (1995). Mitotic forces control a cell-cycle checkpoint.. Nature.

[24] Carpenter AT (1994). Chiasma function.. Cell.

[25] Page SL, Hawley RS (2004). The genetics and molecular biology of the synaptonemal complex.. Annu Rev Cell Dev Biol.

[26] Gerton JL, Hawley RS (2005). Homologous chromosome interactions in meiosis: diversity amidst conservation.. Nat Rev Genet.

[27] Petronczki M, Siomos MF, Nasmyth K (2003). Un menage a quatre: the molecular biology of chromosome segregation in meiosis.. Cell.

[28] Morgan TH (1914). Complete linkage in the second chromosome of the male Drosophila melanogaster.. Science.

[29] Meyer GF, Houwinck AL, Spit BJ (1960). The fine structure of spermatocyte nuclei of Drosophila melanogaster.. Proceedings of the European regional conference on electron microscopy.

[30] Thomas SE, Soltani-Bejnood M, Roth P, Dorn R, Logsdon JM (2005). Identification of two proteins required for conjunction and regular segregation of achiasmate homologs in Drosophila male meiosis.. Cell.

[31] Arya GH, Lodico MJ, Ahmad OI, Amin R, Tomkiel JE (2006). Molecular characterization of teflon, a gene required for meiotic autosome segregation in male Drosophila melanogaster.. Genetics.

[32] Tomkiel JE, Wakimoto BT, Briscoe A (2001). The teflon gene is required for maintenance of autosomal homolog pairing at meiosis I in male Drosophila melanogaster.. Genetics.

[33] Cooper KW, Demerec M (1950). Normal spermatogenesis in Drosophila.. Biology of Drosophila.

[34] Fuller MT, Bate M, Martinez Arias A (1993). Spermatogenesis.. The development of Drosophila melanogaster.

[35] Cenci G, Bonaccorsi S, Pisano C, Verni F, Gatti M (1994). Chromatin and microtubule organization during premeiotic, meiotic and early postmeiotic stages of Drosophila melanogaster spermatogenesis.. J Cell Sci.

[36] Vazquez J, Belmont AS, Sedat JW (2002). The dynamics of homologous chromosome pairing during male Drosophila meiosis.. Curr Biol.

[37] Steffensen S, Coelho PA, Cobbe N, Vass S, Costa M (2001). A role for Drosophila SMC4 in the resolution of sister chromatids in mitosis.. Curr Biol.

[38] Dej KJ, Ahn C, Orr-Weaver TL (2004). Mutations in the Drosophila condensin subunit dCAP-G: defining the role of condensin for chromosome condensation in mitosis and gene expression in interphase.. Genetics.

[39] Savvidou E, Cobbe N, Steffensen S, Cotterill S, Heck MM (2005). Drosophila CAP-D2 is required for condensin complex stability and resolution of sister chromatids.. J Cell Sci.

[40] Bickel SE, Wyman DW, Orr-Weaver TL (1997). Mutational analysis of the Drosophila sister-chromatid cohesion protein ORD and its role in the maintenance of centromeric cohesion.. Genetics.

[41] Ashburner M, Golic KG, Hawley RS (2005). Drosophila: A laboratory handbook.

[42] McKee BD, Lumsden SE, Das S (1993). The distribution of male meiotic pairing sites on chromosome 2 of Drosophila melanogaster: meiotic pairing and segregation of 2-Y transpositions.. Chromosoma.

[43] McKee BD, Karpen GH (1990). Drosophila ribosomal RNA genes function as an X-Y pairing site during male meiosis.. Cell.

[44] McKee BD, Habera L, Vrana JA (1992). Evidence that intergenic spacer repeats of Drosophila melanogaster rRNA genes function as X-Y pairing sites in male meiosis, and a general model for achiasmatic pairing.. Genetics.

[45] Cooper KW (1964). Meiotic conjunctive elements not involving chiasmata.. Proc Natl Acad Sci U S A.

[46] Somma MP, Fasulo B, Siriaco G, Cenci G (2003). Chromosome condensation defects in barren RNA-interfered Drosophila cells.. Genetics.

[47] Baker BS, Carpenter AT, Esposito MS, Esposito RE, Sandler L (1976). The genetic control of meiosis.. Annu Rev Genet.

[48] Baker BS, Carpenter AT (1972). Genetic analysis of sex chromosomal meiotic mutants in Drosophila melanogaster.. Genetics.

[49] Wakimoto BT, Lindsley DL, Herrera C (2004). Toward a comprehensive genetic analysis of male fertility in Drosophila melanogaster.. Genetics.

[50] Hirai K, Toyohira S, Ohsako T, Yamamoto MT (2004). Isolation and cytogenetic characterization of male meiotic mutants of Drosophila melanogaster.. Genetics.

[51] Gethmann RC (1974). Meiosis in male Drosophila melanogaster I. Isolation and characterization of meiotic mutants affecting second chromosome disjunction.. Genetics.

[52] Yu HG, Koshland D (2005). Chromosome morphogenesis: condensin-dependent cohesin removal during meiosis.. Cell.

[53] Hirota T, Gerlich D, Koch B, Ellenberg J, Peters JM (2004). Distinct functions of condensin I and II in mitotic chromosome assembly.. J Cell Sci.

[54] Claycomb JM, MacAlpine DM, Evans JG, Bell SP, Orr-Weaver TL (2002). Visualization of replication initiation and elongation in Drosophila.. J Cell Biol.

[55] Bonaccorsi S, Giansanti MG, Cenci G, Gatti M, Ashburner M, Hawley RS, Sullivan W (2000). Cytological analysis of spermatocyte growth and male meiosis in Drosophila melanogaster.. Drosophila protocols.

[56] Pimpinelli S, Bonaccorsi S, Fanti L, Gatti M, Sullivan W, Ashburner M, Hawley RS (2000). Preparation and analysis of Drosophila mitotic chromosomes.. Drosophila protocols.

[57] Dernburg AF, Sullivan W, Ashburner M, Hawley RS (2000). In situ hybridization to somatic chromosomes.. Drosophila protocols.

[58] Koundakjian EJ, Cowan DM, Hardy RW, Becker AH (2004). The Zuker collection: a resource for the analysis of autosomal gene function in Drosophila melanogaster.. Genetics.

[59] Sweeney SJ, Campbell P, Bosco G (2008). Drosophila sticky/citron kinase is a regulator of cell-cycle progression, genetically interacts with argonaute 1 and modulates epigenetic gene silencing.. Genetics.

