# Supplementary figures and images for: Condensin II Resolves Chromosomal Associations to Enable Anaphase I Segregation in Drosophila Male Meiosis
Source: PLoS Genet. 2008 Oct 17;4(10):e1000228. doi: 10.1371/journal.pgen.1000228 (PMC2562520; doi:10.1371/journal.pgen.1000228)

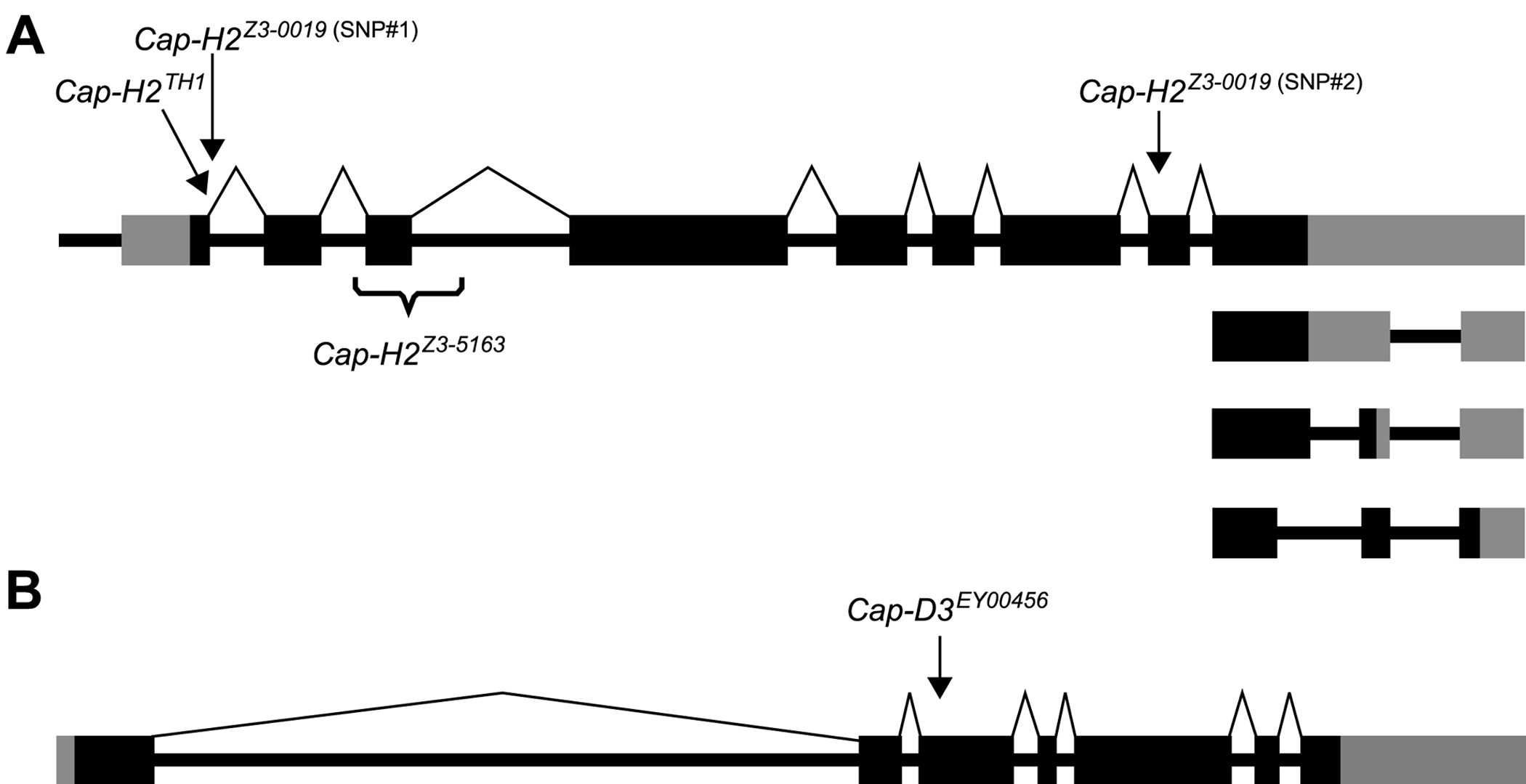

Supplement: Figure S1 — Cap-H2 and Cap-D3 denoting locations of each mutant allele. Coding regions are depicted in black and 5′ and 3′ UTRs in gray. (A) Cap-H2 genomic locus showing splicing patterns found in a Cap-H2 cDNA library. Cap-H2TH1 is a GT to GC alteration in the first intron's splice acceptor site (tgaagaagcggaagcgggt to tgaagaagcggaagcgggc) and was found on the chromosome carrying Df(3L)W10. Cap-H2Z3-0019 carries two SNPs. The first (SNP#1) is an A to T base change in the first intron (gaagcgggtaagcatcca to gaagcgggtaagcatcct) and the second (SNP#2) a G to A mutation changing tagatccgggactgg into tagatccgggactag that switches a tryptophan codon into a stop codon. Cap-H2Z3-5163 is an aberration that has only been defined as to the right of a PstI restriction site ( ctgcagatcctcaaatac) and to the left of a forward primer binding site gttaatggacgatagggcacgtt (as characterized with preliminary southern and PCR analyses) and is consistent with either an insertion or rearrangement. (B) Cap-D3 genomic locus as detailed in the Drosophila melanogaster genome release 4.3. Allele Cap-D3EY00456 is a P-element insertion into the third exon. (6.6 MB TIFF) [file pgen.1000228.s001.tif]
